# Supplementary material for: Nutritional, Cardiovascular Health and Lifestyle Status of ‘Health Conscious’ Adult Vegans and Non-Vegans from Slovenia: A Cross-Sectional Self-Reported Survey
Source: Int J Environ Res Public Health. 2021 Jun 2;18(11):5968. doi: 10.3390/ijerph18115968 (PMC8199727; doi:10.3390/ijerph18115968)
Supplement: Supplementary file 1 [file ijerph-18-05968-s001.zip › ijerph-1222043-supplementary.pdf]

Table S1. Sociodemographic and other characteristics of participants.

**Parameter**	**Vegans** (n = 51)	**Non-vegans** (n = 29)	*p*-value
	n (%)	*n (%)*	
Partner status			0.362
Married	17 (33)	15 (52)	
Extramarital	21 (41)	10 (34)	
Single	9 (18)	2 (7)	
Divorced	4 (8)	2 (7)	
Living environment			0.209
City	16 (31)	5 (17)	
Suburban	20 (39)	10 (34)	
Rural	15 (29)	14 (48)	
Education			0.073
Primary /High School	19 (37)	15 (52)	
College	2 (4)	2 (7)	
Bachelor/university degree	26 (51)	11 (38)	
Masters/PhD degree	4 (8)	1 (3)	
Employment status			0.351
Employed/helping in family business	29 (57)	18 (62)	
Self-employed/contract	11 (22)	7 (24)	
Retired	0	2 (7)	
High-school/student	6 (12)	1 (3)	
Unemployed	4 (8)	1 (3)	
Income (€)			0.230
≤ 501	1 (2)	2 (7)	
501–900	6 (12)	0	
901–1300	6 (12)	7 (24)	
1301–1700	12 (23)	5 (17)	
1701–2100	8 (16)	4 (14)	
≥ 2101	18 (35)	11 (38)	
Smoking			0.071
Current	2 (4)	4 (14)	
Former	9 (18)	9 (31)	
Never	40 (78)	16 (55)	

Chi square test was used; where expected frequency in a cell was less than 5, Fisher's exact test was used.

Table S2. Intakes of food groups in frequencies.

**Parameter**	**Vegans**	**Non-vegans**	
Food groups	Intake frequency†		*p* - value
Liquid			
Water	8.7 ± 0.9	8.6 ± 1.2	0.578
Coffee/tea	6.8 ± 2.1	6.5 ± 2.4	0.566
Fruit/vegetable juice	2.4 ± 1.8	2.6 ± 1.7	0.423
Sport drinks	2.2 ± 1.8	1.9 ± 1.1	0.767
Energy drinks	1.1 ± 0.2	1.2 ± 0.6	0.220
Wine	1.1 ± 0.7	2.8 ± 1.7	**< 0.001**
Beer	1.4 ± 0.6	2.4 ± 1.8	**0.005**
Non-alcoholic beer	1.2 ± 0.4	1.7 ± 1.1	**0.031**
Spirits	1.4 ± 0.5	1.9 ± 1.0	**0.021**
Enriched soy/cereal beverages	2.4 ± 2.3	1.5 ± 1.1	0.089
Unenriched soy/cereal beverages	3.0 ± 2.1	1.7 ± 1.6	**0.003**
Animal-based foods			
Yoghurt/cheese	1.0 ± 0.8	3.3 ± 2.0	**< 0.001**
Milk/chocolate milk	1.1 ± 0.4	3.3 ± 2.0	**< 0.001**
Processed meat products	1.1 ± 0.5	3.3 ± 2.2	**< 0.001**
Offal/liver/kidney	1.0 ± 0.2	1.4 ± 0.6	**< 0.001**
Meat	1.1 ± 0.6	4.6 ± 1.6	**< 0.001**
Fish and products	1.1 ± 0.6	2.7 ± 1.1	**< 0.001**
Eggs	1.1 ± 0.5	4.9 ± 1.5	**< 0.001**
Shellfish	1.0 ± 0.3	1.4 ± 0.8	**0.013**
Butter/lard	1.1 ± 0.3	2.5 ± 1.7	**< 0.001**
Plant-based foods			
Plant-based salads/humus	6.7 ± 2.0	4.7 ± 2.2	**< 0.001**
Meat substitute/soya tofu	5.8 ± 1.6	4.3 ± 1.8	**< 0.001**
Pasta	4.5 ± 1.0	4.5 ± 1.0	0.725
Rise/other cereals	6.6 ± 1.1	4.9 ± 0.8	**0.005**
White bread/products	1.9 ± 1.1	3.0 ± 2.3	0.068
Whole grain bread/products	6.9 ± 1.4	5.5 ± 1.5	**0.265**
Baked potatoes	4.1 ± 1.0	3.8 ± 1.2	0.288
Boiled/puree potatoes	4.4 ± 1.0	3.9 ± 1.2	0.097
Fried potatoes/croquettes	1.1 ± 0.3	1.7 ± 1.1	**< 0.001**
Legumes	8.1 ± 1.0	4.7 ± 1.3	**< 0.001**
Raw vegetables	7.9 ± 1.2	6.4 ± 1.2	**< 0.001**
Boiled vegetables	7.0 ± 1.0	6.1 ± 1.0	**< 0.001**
Fruits	8.6 ± 1.2	6.9 ± 1.0	**< 0.001**
Dry fruits	3.8 ± 2.1	3.5 ± 1.8	0.637
Seeds	7.4 ± 1.5	5.2 ± 2.1	**< 0.001**
Nuts	5.8 ± 1.7	4.7 ± 1.6	**0.011**
Sweets			
Chocolate/candy	1.5 ± 1.4	3.4 ± 1.8	**0.016**
Pastries	2.3 ± 1.1	2.9 ± 1.2	**0.041**
Confectionery/croissants/cakes	1.7 ± 1.0	2.5 ± 1.1	**< 0.001**
Others			
Ketchup	1.7 ± 1.1	1.7 ± 1.1	0.927
Margarine	1.0 ± 0.0	1.3 ± 0.9	**0.007**
Dressings	1.1 ± 0.3	1.5 ± 0.9	**0.023**

Data are means ± SD. Statistically significant values are written bold. †A higher number means more often; 1 - never, 9 - more than 3 times per day. For all variables, Mann-Whitney U-tests were used.

Table S3. Transportation, everyday sitting, and PA of vegans and non-vegans.

	L-IPAQ score		
**Parameter**	**Vegans**	**Non-vegans**	p - value
Transportation (min/day)[†]	19 ± 15	22 ± 36	0.269
Weekly sitting (h/day)	5.4 ± 2.6	4.2 ± 3.2	0.063
Weekend sitting (h/day)	3.6 ± 1.9	4.9 ± 5.8	0.159[*]
Walking PA (MET min/week)	2045 ± 2500	2547 ± 2871	0.172
Moderate-intensity PA (MET min/week)	2120 ± 1400	3200 ± 1680	**0.045**
Vigorous-intensity PA (MET min/week)	2160 ± 800	2400 ± 1056	0.324
Total (MET min/week) score	6325 ± 4870	8147 ± 4392	0.192

Data are means ± SD. Statistically significant values are written in bold. [†]Transport related to arrival and departure to work (i.e., with car, a train, by bus, using bicycle or by walking). [*]A t-test was used; for other variables, Mann-Whitney U-tests were used.

Table S4. Sleep quality and patterns of vegans and non-vegans.

Parameter	PSQI score Vegans	Non-vegans	*p*-value
Component 1	Subjective sleep quality		
	0.6 ± 0.6	0.9 ± 0.6	0.147
Component 2	Sleep latency		
	0.5 ± 0.8	0.5 ± 0.8	0.986
Component 3	Sleep duration		
	0.7 ± 0.7	0.8 ± 0.7	0.621
Component 4	Sleep efficiency		
	0.1 ± 0.4	0.0 ± 0.2	0.289
Component 5	Sleep disturbance		
	0.9 ± 0.4	0.9 ± 0.4	0.785
Component 6	Sleep medication use		
	0	0.1 ± 0.6	0.185
Component 7	Daytime dysfunction		
	0.3 ± 0.6	0.8 ± 1.0	**0.009**
Global sleep quality†	3.2 ± 1.9	4.0 ± 2.3	0.139

Data are mean ± SD. †Sum of seven component scores (range: 0–21; ≤ 5 indicates good sleep quality, >5 indicates poor sleep quality). Statistically significant values are written in bold. For all variables, Mann-Whitney U-tests were used.

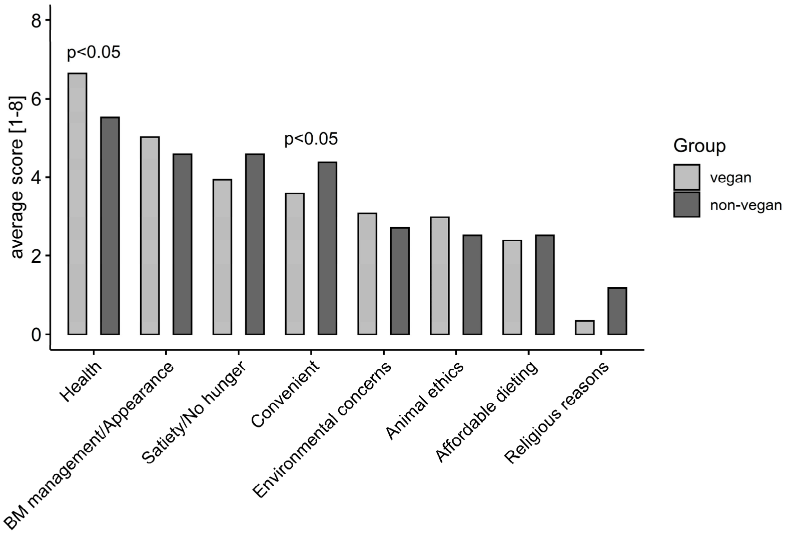

Figure S1. Motives of vegan and non-vegan for adopting dietary pattern.

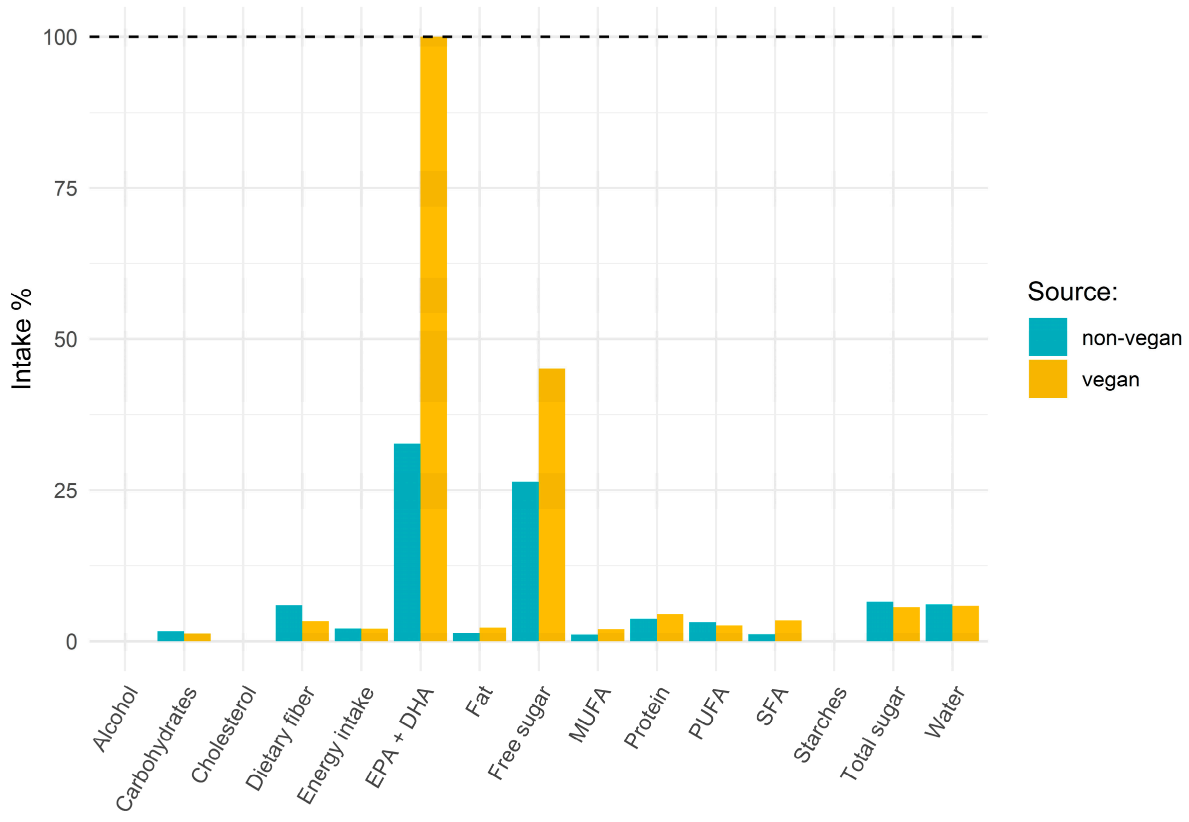

Figure S2. Proportion of macronutrients from supplementation.

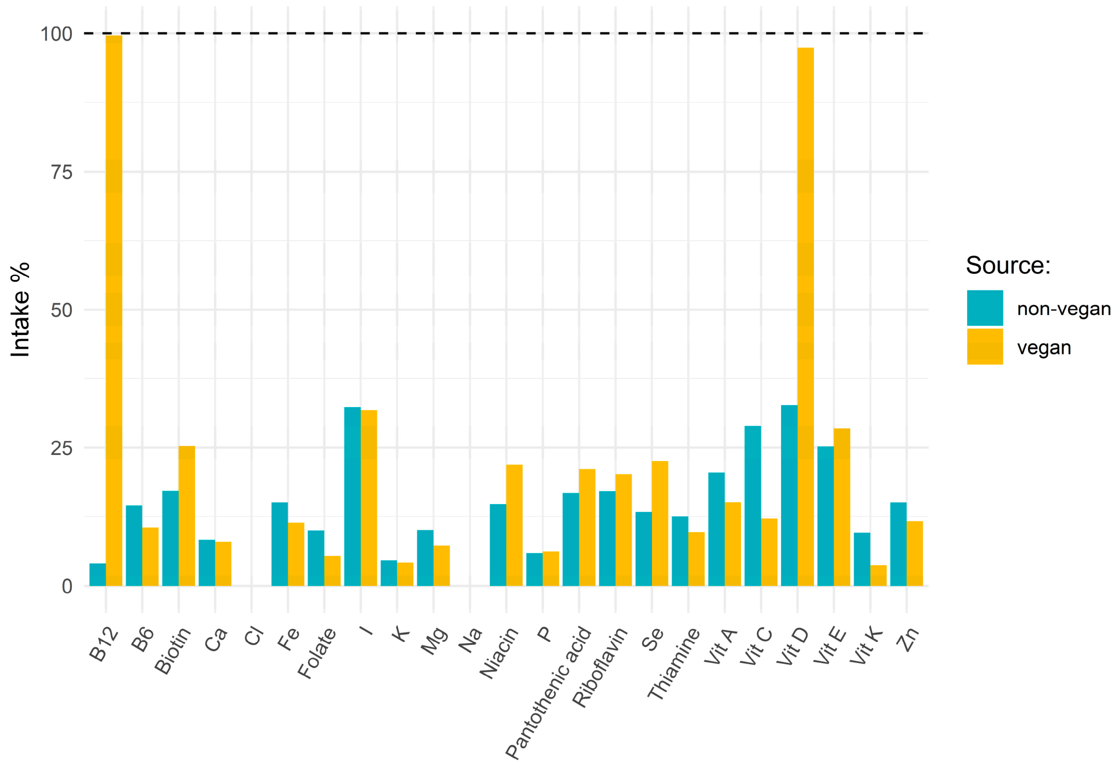

Figure S3. Proportion of micronutrients from supplementation.
